# Use of glatiramer acetate between 2010–2015: effectiveness, safety and reasons to start GA as first or second line treatment in Swiss multiple sclerosis patients

**DOI:** 10.1186/s12883-019-1383-6

**Published:** 2019-07-12

**Authors:** Chiara Zecca, Giulio Disanto, Rosaria Sacco, Gianna C. Riccitelli, Claudio Gobbi

**Affiliations:** 1 Neurocenter of Southern Switzerland, Ospedale Regionale di Lugano, Via Tesserete 46, 6903 Lugano, Switzerland; 2 Faculty of biomedical Sciences, Università della Svizzera Italiana, Lugano, Switzerland; 3 Neuroimaging Research Unit, Institute of Experimental Neurology, Division of Neuroscience, San Raffaele Scientific Institute, Vita-Salute San Raffaele University, Milan, Italy

**Keywords:** CIS, GA, Glatiramer acetate, Multiple sclerosis, Relapsing remitting, Therapy

## Abstract

**Background:**

Glatiramer acetate (GA) is one of the first therapies approved for multiple sclerosis (MS). We prospectively included and monitored drug-naïve and pre-treated MS patients who had been prescribed GA for 1 year, to investigate reasons for GA prescription, its effectiveness and safety in real life clinical practice.

**Methods:**

One year, prospective, multicentre, observational study performed between 2010 and 2015 in consecutive MS and clinically isolated syndrome patients starting GA as a first (“naïve”) or second (“switcher”) line therapy. Primary endpoint was the annualized relapse rate (ARR) over 1 year of GA treatment (from baseline, V1, to 12 months, V2) in naïve and switchers compared to previous 24 months. Secondary endpoints were: EDSS changes between V1 and V2, frequency of adverse events, and reasons for prescribing and discontinuing GA. Baseline demographics and clinical characteristics were retrieved from medical records, and outcome measures were documented at V1 and V2, and in case of clinical worsening.

**Results:**

One hundred ninety-four consecutive patients were monitored over 12 months (*N* = 64 naïve, *N* = 130 switchers). Side effect profile (naïve = 36%, switchers = 28%) and comorbidities (naïve = 31%, switchers = 15%) were the most frequent reasons to start GA. The ARR was reduced in both naïve and switchers during V1–2 as compared to the 24 months preceding V1 [naïve: 0.0 (0.0–0.0) vs 0.5 (0.5–1.0, *p* = 2.9e-10); switchers: 0.0 (0.0–0.0) vs 0.5 (0.0–0.5, *p* = 0.022)]. EDSS at V2 was significantly reduced only in naïve [(1.5 (1.0–2.5) vs 2.0 (1.5–2.5), *p* = 0.003)]. Naïve status and EDSS at V1 were negatively associated with EDSS change between V1-V2 in multivariable analysis (regression coefficient = − 0.436, *p* = 0.008, and regression coefficient = − 0.263, *p* = 6.18e-05, respectively). No new unexpected AE was reported.

**Conclusion:**

In our Swiss cohort, GA was prescribed mainly to naïve or switcher MS patients fearing interferon related side effects, with various comorbidities or considering pregnancy, and showed effectiveness and safety comparable with data of previous GA studies.

**Electronic supplementary material:**

The online version of this article (10.1186/s12883-019-1383-6) contains supplementary material, which is available to authorized users.

## Background

Glatiramer acetate (GA, Copaxone®) is a synthetic amino acid polymer analogue of myelin basic protein and represents one of the first approved therapies for multiple sclerosis (MS) worldwide. It is now believed to exert its mechanism of action mainly through immune modulation leading to reduced central nervous system inflammation [1]. Its efficacy and safety profile in relapsing remitting MS (RRMS) have been investigated and confirmed by long-term studies up to 22 years, with more than two million patient-years exposure [2, 3]. Its indication was extended to the treatment of clinically isolated syndromes (CIS) following the results of the PRECISE trial [4], and more recently a new administration regimen (i.e. GA 40 mg 3 times a week) has been approved as an alternative option with advantages in terms of injection frequency [5].

Several new drugs including oral compounds and monoclonal antibodies have been approved for MS treatment more recently. Despite the lack of head to head trials, their efficacy is often considered superior to GA (especially for monoclonal antibodies), at the expense of a less favourable short and long time safety profile [6–11]. Since their introduction, these new compounds have expanded the MS therapeutic landscape and changed treatment algorithms. It is now less clear which patients are still likely to benefit from GA treatment. We therefore aimed at characterizing Swiss MS patients that started with GA treatment in real life clinical practice, particularly assessing the reasons why GA was currently prescribed, as well as its effectiveness and safety, and if these differ between patients using GA as first or second line MS therapy.

## Methods

### Ethics approval and consent to participate

This research is a field report from practice and did not require ethical approval when it was started, long before the new ordinance on research in humans came into force (01.01.2014). A simple notification to the ethics committee of each Canton (Ethics Committe northwest/central Switzerland for the Cantons Aargau, Basel, Jura, Lucerne, Unterwalden, Schwyz, Uri, Zug; Ethics Committee Bern for the Cantons Bern, Fribourg, Valais; Ethics Committee Geneva for the canton Geneva, Ethikkommission Ostschweiz for the Cantons Appenzell, Sanct Gallen, Thurgau; Ethics Committee Ticino for the Canton Ticino; Ethics Committee Vaud for the Cantons Fribourg, Neuchâtel, Vaud, Valais; Ethics Committee Zurich for the Cantons Glarus, Graubünden, Schaffhausen, Zurich), in which the various participating centers were located, was sufficient and did not receive a reference number. The funding body notified these practice experience observations to the ECs to ease the administrative burden of the coordinating and all participating centers. All participants were adults and signed a written informed consent regarding the use of their health care data for the purpose of this research.

### Design

This was a prospective, multicentre, observational study on patient data to reflect real life clinical practice on the prescription and use of GA across 45 hospital and office-based neurologists in Switzerland between 2010 and 2015.

### Aims

The study aimed at 1) investigating GA effectiveness and safety when used as either first line treatment in previously untreated patients or as second line in patients switching from another treatment; 2) assessing the reasons for choosing GA among different MS therapies.

### Patient population

All patients diagnosed with RRMS and CIS according to McDonald 2005 [12] who were prescribed GA according to the approved Swiss label between 2010 and 2013 and during 2015 at participating centers were consecutively included for monitoring within ±30 days since GA start. Patients were stratified according to previous MS therapy: those who received GA as a first line drug for MS constituted the “naïve group”, whilst patients who started GA after discontinuing a different immune therapy were included in the “switcher group”.

### Endpoints

The primary endpoint was the annualized relapse rate (ARR) over 1 year of GA treatment (from baseline, V1, to 12 months, V2) in naïve and switching patients compared to the ARR of the previous 24 months. Secondary endpoints were EDSS changes over the study period, the frequency and characterization of adverse events (AEs) occurring between V1 and V2, and the frequencies of reasons for prescribing or discontinuing GA according to pre-defined lists (see “clinical assessment”).

### Clinical assessment

Treating neurologists collected in a case report form (CRF) patients’ demographics at V1 and clinical characteristics at V1 and after 12 months (visit 2, V2). Past MS history was retrieved from medical records.

The reasons for starting with or switching to GA were recorded by the treating neurologists in the CRF, referring to the following pre-defined list of categories: 1. Avoid flu–like syndrome/favourable side effect profile; 2. Concomitant depression/fatigue/cognitive problems; 3. Comorbidities; 4. Low disease activity; 5. Pregnancy planning; 6. JCV+ status; 7. Patient’s choice; 8. Others.

At baseline (V1), patients were advised to inform their treating neurologist in case of new neurological symptoms, any new general symptoms, or treatment discontinuation. If these occurred, the treating neurologist examined patients within 2 weeks to assess the occurrence of relapses and/or adverse events, or to establish the reason for treatment discontinuation. The treating neurologists additionally examined patients between V1 and V2 according to their clinical practice.

Relapses were defined according to international diagnostic and therapeutic guidelines as newly developing neurological symptoms or reactivation of pre-existing neurological deficits for a minimum of 24 h in the absence of an increase in body temperature or infections occurring at least 30 days after the preceding episode [13].

Disability was measured by the Expanded Disability Status Scale (https://www.neurostatus.net/) [14].

AEs occurring during the study were noted in the CRF and classified as local reactions, systemic reactions or others. The treating neurologist also specified a possible causal role of GA treatment in the occurrence of the AEs. Reasons to discontinue GA were classified as follows: 1. Patient’s choice; 2. Treatment related side effects; 3. Treatment felt to be ineffective by the patient; 4. Adverse events; 5. Treatment failure; 6. Pregnancy; 7. Other reasons. The immune modulating therapies started after GA discontinuation and the reasons why these were chosen were also reported according to this classification: 1. breakthrough disease activity, 2. adverse events; 3. others.

The following data were collected in the CRF by the treating neurologists during V1 and V2:

- V1 (baseline): MS subtype, disease onset, number of relapses in the past 2 years, previous immune-modulating therapies and reason for discontinuing, EDSS score at V1, comorbidities and concomitant medications.
- V2 (month 12): MS subtype, number of relapses occurred since V1, EDSS score at V2, adverse events, comorbidities and concomitant medications.

All patients completing V1 and V2 were considered for the analyses.

### Statistics

Continuous and ordinal variables were described by median and interquartile ranges (IQR), categorical variables by counts and percentages. Categorical variables at baseline and during the study were compared using Chi Square (χ^2^) test, continuous and ordinal variables were compared using the non-parametric Mann-Whitney (MW) test for unpaired groups and the Wilcoxon matched (WM) test for paired groups. The annualized relapse rate (ARR) and the EDSS change between V1 and V2 were also compared between naïve and switchers using Poisson and linear regression models respectively, with and without correction for covariates of interest (gender, age, disease duration, ARR in the previous 24 months (24 M) and EDSS at V1). Residuals were checked for normality under linear models using histograms and plots of residuals vs fitted values. To further correct for baseline imbalances, naïve and switcher patients were matched 1:1 by using propensity scores (PS), based on gender, age, disease duration, ARR in the previous 24 months (24 M) and EDSS at V1. A conservative caliper size of 0.1 standard deviations of the logit of the PS was used to provide adequate matching. Significance level was set at *p* = 0.05. All analyses were performed using R (https://www.r-project.org/) and the R package “non-random”.

## Results

### Baseline characteristics

Two hundred and twenty-six patients were recruited in the study. Thirty-two patients did not complete V2 (lost to follow up) and were therefore excluded from following analyses. Notably, there were no significant differences in terms of baseline variables between included and excluded patients (Additional file 1). The characteristics of the included 194 patients are reported in Table 1. One hundred and thirty patients were naïve, 64 switchers. Sixty percent of the switchers had been treated with interferons before GA start. Median (IQR) time interval between discontinuation of prior therapy and the beginning of GA was 4.5 (2.0–5.5) weeks. Naïve patients showed several characteristics that were different from those of the switchers. As compared to switchers, naïve patients were indeed significantly younger (36.0 (29.0–45.0) vs 41.5 (33.0–48.2)), had a larger proportion of CIS (24.6% vs 6.2%) and a shorter disease duration (1.0 (0.0–5.0) vs 6.0 (3.0–13.0) years) (Table 1). As expected, EDSS at V1 was higher in switchers than in naïve patients (2.5 (2.0–3.8) vs 2.0 (1.5–2.5)), while the ARR in the 24 months preceding V1 was higher in naïve than in switchers (0.5 (0.5–1.0) vs 0.5 (0.0–1.5) respectively) (Table 1).

**Table 1:** Baseline variables of switchers and naïve patients and statistical comparison using the χ^2^ or the MW test

Variables	Switchers (*n* = 64)	Naïve (*n* = 130)	*p*
Age [years (IQR)]	41.5 (33.0–48.2)	36.0 (29.0–45.0)	0.014
Sex			
M [n (%)]	10 (15.6)	28 (21.5)	0.496
F [n (%)]	47 (73.4)	92 (70.8)	
NA [n (%)]	7 (10.9)	10 (7.7)	
Disease course			
CIS [n (%)]	4 (6.2)	32 (24.6)	0.003
RRMS [n (%)]	60 (93.8)	98 (75.4)	
Last treatment			
INF [n (%)]	38 (59.4)	NA	
FTY [n (%)]	5 (7.8)	NA	
DMF [n (%)]	3 (4.7)	NA	
NTZ [n (%)]	11 (17.2)	NA	
MTX [n (%)]	1 (1.6)	NA	
NA [n (%)]	6 (9.4)	NA	
Age at disease onset [years (IQR)]	31.0 (24.0–39.5)	31.0 (26.0–40.0)	0.662
Disease duration [years (IQR)]	6.0 (3.0–13.0)	1.0 (0.0–5.0)	1.50E-08
ARR previous 24 months	0.5 (0.0–0.5)	0.5 (0.5–1.0)	0.005
EDSS at V1 [score (IQR)]	2.5 (2.0–3.8)	2.0 (1.5–2.5)	0.0006
V1 - GA treatment [days] (IQR)]	0.5 (−2.2–16.7)	3.0 (− 7.0–16.0)	0.961

*ARR* Annualized relapse rate, *DMF* dimethylfumarate, *EDSS* Expanded diability status scale, *FTY* fingolimod, *GA* Glatiramer acetate, *INF* interferone, *IQR* interquartile range, *MTX* mitoxantrone, *NTZ* Natalizumab

### Longitudinal changes in ARR and EDSS after GA start in naïve and switchers

**Fig. 1:**
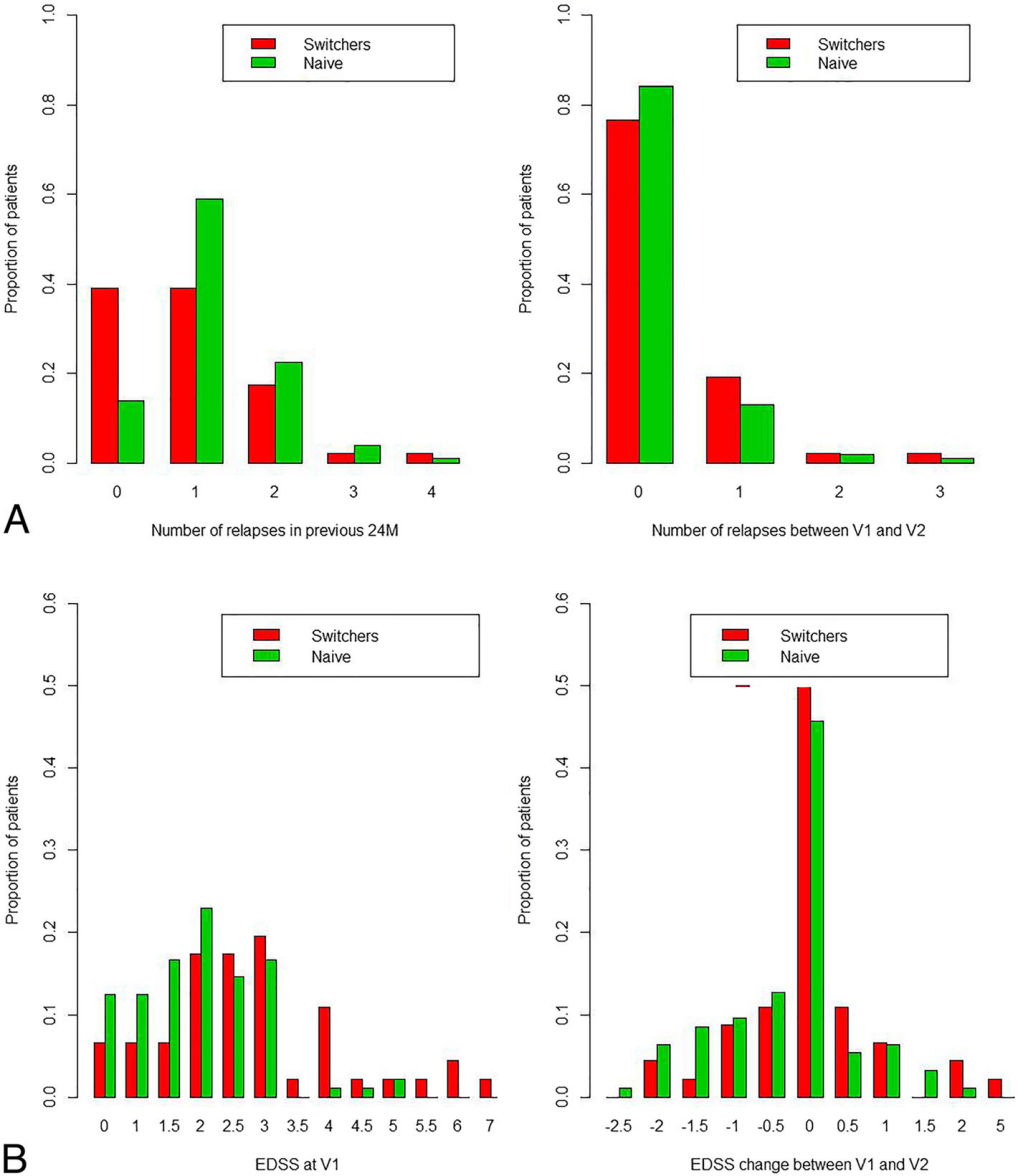
**a** Number of relapses in the 24 months preceding V1 (left) and between V1 and V2 in naïve (green) and switchers (red) (right). **b** Expanded disability status score (EDSS) at V1 (left); EDSS change between V1 and V2 in naïve (green) and switchers (red) (right)

The time between V1 and V2 was similar between naïve and switcher patients (356 (316–384) vs 360 (335–383) days respectively, *p* = 0.978). At V2, 89 (68.5%) patients in the naïve and 43 (67.2%) in the switcher group were still on GA treatment (*p* = 0.988). The number of naïve patients experiencing 0, 1, 2 and 3 relapses between V1 and V2 was 111 (85.4%), 16 (12.3%), 2 (1.5%) and 1 (0.8%). The number of switchers experiencing 0, 1, 2 and 3 relapses between V1 and V2 was 51 (79.7%), 11 (17.2%), 1 (1.6%) and 1 (1.6%). The ARR was significantly reduced in the V1-V2 period as compared to the 24 months preceding V1 in both naïve (0.0 (0.0–0.0) vs 0.5 (0.5–1.0), respectively, *p* = 2.9e-10) and switchers (0.0 (0.0–0.0) vs 0.5 (0.0–0.5), respectively, *p* = 0.022) (Table 2, Fig. 1). Similarly, the EDSS was significantly reduced at V2 as compared to V1 in naïve patients (1.5 (1.0–2.5) vs 2.0 (1.5–2.5), respectively, *p* = 0.003). In contrast, EDSS scores were similar at V1 and V2 in the switchers (2.5 (2.0–3.8) vs 2.5 (1.5–4.0), respectively, *p* = 0.852) (Table 2, Fig. 1).

**Table 2:** Longitudinal changes in ARR and EDSS in naïve and switcher patients during V1-V2 tested using the WM test

Pre vs Post comparison	Previous 24 months	V1-V2	*p*
Switchers, *n* = 130			
ARR	0.5 (0.0–0.5)	0.0 (0.0–0.0)	0.022
Naïve, *n* = 64			
ARR	0.5 (0.5–1.0)	0.0 (0.0–0.0)	2.90E-10
	V1	V2	*p*
Switchers, *n* = 130			
EDSS	2.5 (2.0–3.8)	2.5 (1.5–4.0)	0.852
Naïve, *n* = 64			
EDSS	2.0 (1.5–2.5)	1.5 (1.0–2.5)	0.003

*ARR* Annualized relapse rate, *EDSS* Expanded disability status scale, *V* Visit

### Response to GA treatment in naïve vs switchers and associations with other covariates

We tested several variables including naïve vs switcher status for association with ARR during V1-V2 and EDSS change between V1 and V2 using linear regression models. In univariable analyses, naïve status was negatively associated with EDSS change between V1 and V2 (regression coefficient = − 0.296, *p* = 0.042). This association remained significant (regression coefficient = − 0.436, *p* = 0.008) when gender, age, disease duration, ARR in the previous 24 months and EDSS at V1 were included in the same multivariable model (Table 3). EDSS at V1 was also negatively associated with EDSS change between V1 and V2 (regression coefficient = − 0.263, *p* = 6.18e-05). No other statistically significant associations were present. Similar significant associations were present in the restricted group of PS matched naïve (*N* = 53) and switcher (*N* = 53) patients (naïve status: regression coefficient = − 0.649, *p* = 0.001; EDSS at V1: regression coefficient = − 0.276, *p* < 0.001).

**Table 3:** Regression models testing the association between occurrence of relapses during V1 and V2 (Poisson model) as well as EDSS change between V1 and V2 (linear model) with naïve vs switcher status and other covariates

Predicted	Predictors	Regression coefficient	*p*
Relapses V1-V2	Naïve vs Switcher	−0.587	0.164
	Gender (F)	−0.500	0.225
	Age	−0.051	0.016
	Disease duration	−0.020	0.566
	ARR last 24 M	0.077	0.855
	EDSS V1	0.179	0.279
EDSS V1-V2 change	Naïve vs Switcher	−0.436	0.008
	Gender (F)	−0.081	0.641
	Age	0.007	0.308
	Disease duration	0.001	0.927
	ARR last 24 months	−0.297	0.094
	EDSS V1	−0.263	6.18E-05

*ARR* Annualized relapse rate, *EDSS* Expanded disability status scale, *V* Visit

There was no association between naïve status (vs. switchers) and occurrence of relapses between V1 and V2 in either univariable (regression coefficient = − 0.345, *p* = 0.288) or multivariable analyses (regression coefficient = − 0.587, *p* = 0.164). A significant negative association was present between age and occurrence of relapses (regression coefficient = − 0.051, *p* = 0.0163) (Table 3). This was also the only significant association present in the restricted group of PS matched patients (regression coefficient = − 0.076, *p* = 0.004).

### Factors motivating the start of GA therapy

To avoid flu-like symptoms and more generally the favourable side effect profile of GA were the most frequent reason to prescribe GA in both naïve (36%) and switcher (28%) patients. In the naïve group fatigue, cognitive impairment and depression accounted for 31% of GA prescriptions, while this figure was 15% for the switcher group. In the latter, comorbidities motivated 17% of GA prescriptions. Pregnancy planning represented a reason to start GA in 7% of naïve and 8% of switcher patients. In the switcher group, positive JC virus status accounted for 13% of prescriptions. GA was started in 8% of naïve patients because of patients’ choice; this was not the case for any patient in the switcher group (Table 4).

**Table 4:** Main factors motivating the start of a treatment with glatiramer acetate

Reason to start glatiramer acetate	Overall *n* = 145 (%)	Naïve *n* = 92 (%)	Switchers *n* = 53 (%)
Avoid flu–like syndrome / side effect profile	48	33 (36)	15 (28)
Depression / fatigue / cognitive problems	37	29 (31)	8 (15)
Comorbidities	13	4 (4)	9 (17)
Low disease activity	11	7 (8)	4 (8)
Pregnancy planning	10	6 (7)	4 (8)
JCV+ status	8	1 (1)	7 (13)
Patient’s choice	7	7 (8)	0
Others	11	5 (5)	6 (11)

### Safety

Twenty-eight adverse events were reported by 26 (13%) of the patients. The most frequent were injection site reactions (*N* = 7) and relapses motivating hospitalization (*N* = 4). There were two cases of generalized lymphadenopathy, and no unexpected side effects occurred (Additional file 2). Sixty-two patients discontinued GA before V2. The most frequent reasons for discontinuing were patient’s choice (*N* = 22), treatment failure (*N* = 11) and AEs (*N* = 8). (Additional file 3). Eight patients reported pregnancies during GA therapy. GA was discontinued in all cases within the first 12 weeks of pregnancy. No complications or relapses were reported during pregnancies, nor during breastfeeding (5/8 patients). All babies were healthy at birth. GA was resumed after delivery in 1 patient and after breastfeeding in 4 patients. Two patient started dimethyl-fumarate after delivery and breastfeeding, respectively, and 1 started fingolimod after delivery. For 44 patients, the immune therapy after GA discontinuation was reported. The most frequently prescribed therapies were natalizumab (*N* = 12, 27%; *N* = 10 breakthrough disease activity,), fingolimod (*N* = 12, 27%; *N* = 8 breakthrough disease activity, *N* = 2 AEs), and interferon beta 1a (*N* = 10, 23%; *N* = 6 AEs). Six (13%) patient were prescribed with teriflunomide, and 4 (10%) with dimethyl-fumarate, (AEs in all cases).

For the remaining 18 patients with similar baseline characteristics (data not shown) information on the immune therapy after GA discontinuation was not available.

## Discussion

Our observational, prospective study describes the reasons for prescription of GA in real life clinical practice and monitored its efficacy and safety over a 1-year period in a contemporary MS population in Switzerland (2010–2015).

Thirty-six percent of naïve and 28% of switchers started GA because of absence of flu-like symptoms and the favourable safety profile. Our findings are in line with several studies highlighting the relevance of intolerable drug-associated side effects among reasons that motivate MS therapy change [13–15]. Importantly GA does not negatively influence fatigue and mood disorders [15, 16], liver dysfunction is rare and no major drug-to-drug interactions are present. Pregnancy exposure to GA in women with MS is probably acceptable [17], and 7% of naïve and 8% of switchers were indeed started with GA because of pregnancy planning. Our study therefore further highlights how drug-associated side effects represent a major driver in the choice of MS therapy in both naïve patients and in those who have already experienced other MS treatments.

The rate of relapses was very low in the year following GA initiation and significantly reduced as compared to the 2 years before GA start. Disability scores remained stable in the switcher group and significantly decreased in naïve patients.

Another recent, epidemiological study [18] including Danish patients treated with teriflunomide and dimethyl fumarate in a real world setting found similarly low rates of relapses, probably reflecting the current use of injectables or oral first line therapies in patients with less severe disease activity.

Our results suggest that both naïve and switcher patients can benefit from GA therapy, at least in the 1 year following treatment start. Similarly, another recent, prospective, observational study, involving 754 German patients, showed that GA reduced ARR and disability progression in both naïve and switcher individuals (ARR in switchers: from 0.98 to 0.54, *p* = 0.001; ARR in naïve: from 0.81 to 0.48; *p* = 0.001; EDSS change in switchers: − 0.02 ± 1.12, *p* = 0.86; EDSS change in naïve: − 0.04 ± 1.05, *p* = 0.27) [19]. The difference in absolute ARR values during GA treatment between our study and the study by Ziemmsen and coworkers [20] are most likely due to the differences between the two populations included. Of note, less than 30% of the switchers in our and in the German cohort experienced previous treatment failure under interferons. This and the possible bias deriving from a regression to the mean effect likely explain the less positive results reported in other studies of patients switching between different first line therapies after treatment failure [20]. Our findings suggest that a “lateral” switch could be considered in case of reasons other than treatment failure (e.g. adverse reactions, tolerability issues, comorbidities, pregnancy), while in the case of disease reactivation escalation approaches appear more appropriate [15, 21].

When using linear regression analyses, a significant negative association between naïve status (vs. switchers) and EDSS change between V1 and V2 was observed in both univariable and multivariable models. We believe that the negative association between EDSS change and naïve status (i.e. EDSS tendency to decrease in naïve patients) is likely driven by recovery from recent relapses leading to diagnosis and first MS treatment. We think that this hypothesis is further confirmed by the relapse rate preceding GA start being higher in the naïve than in the switcher group, thus being more affected by regression to the mean effect, and also by a high EDSS at V1 negatively associated with EDSS change at V2 (i.e. patients with high EDSS due to recent relapses are more likely to experience EDSS improvement). However, it is also possible that the larger compensatory neuronal reservoir available in “early” patients could make naïve individuals benefit from GA more than those with a more advanced disease. Nonetheless, that early use of MS therapies is associated with better outcomes has already been shown in other studies [4, 22–24]. No association was instead present between occurrence of relapses and naïve status. However, younger patients appeared to be at higher risk of experiencing relapses, confirming that particular attention should be reserved to patients in their earliest stages of disease.

Concerning safety, GA was overall well tolerated in our study and the proportion of patients reporting adverse events (13%) was in line with findings from similar cohorts. Ziemssen et al. [15] reported that 15.5% of 672 patients who were switched from another therapy to GA, had at least one adverse event during the first year of treatment. Similarly, in the QualiCOP study, 19.1% of patients starting GA had at least one adverse event during a mean follow up of 17.5 months [19]. The overall number of AEs was instead probably underestimated due to study designs, having only one 12 month-, pre-planned visit. This might have prevented our study to capture mild and/or transient AEs, particularly injection related reactions. One-third (62/194) of our patients discontinued GA before study completion, but only a minority of them due to treatment failure or adverse events (6 and 4% of overall patients, respectively). Similarly, after a mean follow up period of 1.5 years, 6.3 and 4.6% of patients discontinued GA because of lack of efficacy and adverse events, respectively, in the COPTIMIZE study [15]. While 9% of patients in the QualiCOP study interrupted GA because of adverse events, no figures are provided for those stopping GA because of treatment failure [19]. Eight uncomplicated pregnancies in our study population contributed further to the body of evidence that continuing GA until conception is safe and might help preventing disease reactivation as opposed to interrupting MS therapy before conception [17, 25].

The main limitation of our study is the lack of radiological data generally more sensitive to disease activity in the short term (i.e. 1 year follow up) as compared to relapses or changes in disability scores. Nonetheless, exactly because the study duration is relatively short and the onset of GA therapeutic effect might take up to 6 months, a 1 year follow up MRI only might have been elusive without an additional scan performed a few months after GA start (i.e. a re-baseline scan). This was not being routinely performed in clinical practice when this observational study was performed. A second limitation is the lack of a control group preventing definitive conclusions about safety and efficacy. However, our primary aim was to characterize Swiss MS patients that are currently prescribed with GA and assess the reasons for choosing GA among different MS therapies rather than its efficacy and the safety. A third limitation of our study is the proportion of patients lost to follow up (14%). While this group of patients might have influenced significantly the results, their baseline characteristics were similar to those of remaining patients, making this hypothesis less likely. Also, we attempted to define predictors of response to GA by including several variables in the regression models with occurrence of relapses and EDSS change as outcome variables. However, this cohort does not represent the appropriate settings where to investigate predictors of response to GA due to the overall reduced sample size and small proportion of patients experiencing disease activity. Finally, our study did not include patients treated with biosimilars of GA, which were not yet approved in Switzerland during the study period.

We believe that our study nevertheless brings important information, as it reflects how the use of GA in daily clinical practice has developed in the most recent years in the setting of several Swiss tertiary level-centers and office-based neurologists.

## Conclusion

While the recent development of several new drugs has considerably improved current MS treatments (especially in patients with severe disease forms), the results of this study suggest that GA is an option for MS treatment, especially when individuals fear interferon related side effects, carry relevant comorbidities or plan a pregnancy. Its clinical efficacy and safety in naïve patients and in those previously treated with other compounds seem to be comparable to findings in previous studies.

## Additional files

Additional file 1: Comparison between the baseline characteristics of patients monitored (V1 V2) and those who had no data on V2 (noV1 V2). (DOCX 15 kb)

Additional file 2: Adverse events reported during study period. (DOCX 14 kb)

Additional file 3: Reasons for discontinuing GA treatment before V2 (*N* = 62). (PDF 572 kb)

## Data Availability

The datasets used and/or analysed during the current study are available from the corresponding author on reasonable request.

## Abbreviations

ARR: Annualized relapse rate
CIS: Clinically isolated syndromes
EDSS: Expanded Disability Status Scale
GA: Glatiramer acetate
IQR: Interquartile ranges
JCV: John cunningham virus
MS: Multiple sclerosis
MW: Mann-Whitney
RRMS: Relapsing remitting multiple sclerosis
V: Visit
WM: Wilcoxon matched

## References

[1] Caporro M, Disanto G, Gobbi C, Zecca C (2014). Two decades of subcutaneous glatiramer acetate injection: current role of the standard dose, and new high-dose low-frequency glatiramer acetate in relapsing-remitting multiple sclerosis treatment. Patient Prefer Adherence.

[2] Boster AL, Ford CC, Neudorfer O, Gilgun-Sherki Y (2015). Glatiramer acetate: long-term safety and efficacy in relapsing-remitting multiple sclerosis. Expert Rev Neurother.

[3] Scott LJ (2013). Glatiramer acetate: a review of its use in patients with relapsing-remitting multiple sclerosis and in delaying the onset of clinically definite multiple sclerosis. CNS drugs.

[4] Comi G, Martinelli V, Rodegher M, Moiola L, Bajenaru O, Carra A, Elovaara I, Fazekas F, Hartung HP, Hillert J (2009). Effect of glatiramer acetate on conversion to clinically definite multiple sclerosis in patients with clinically isolated syndrome (PreCISe study): a randomised, double-blind, placebo-controlled trial. Lancet (London, England).

[5] Wolinsky JS, Borresen TE, Dietrich DW, Wynn D, Sidi Y, Steinerman JR, Knappertz V, Kolodny S (2015). GLACIER: an open-label, randomized, multicenter study to assess the safety and tolerability of glatiramer acetate 40 mg three-times weekly versus 20 mg daily in patients with relapsing-remitting multiple sclerosis. Mult Scler Relat Disord.

[6] Cohen JA, Coles AJ, Arnold DL, Confavreux C, Fox EJ, Hartung HP, Havrdova E, Selmaj KW, Weiner HL, Fisher E (2012). Alemtuzumab versus interferon beta 1a as first-line treatment for patients with relapsing-remitting multiple sclerosis: a randomised controlled phase 3 trial. Lancet (London, England).

[7] Gold R, Kappos L, Arnold DL, Bar-Or A, Giovannoni G, Selmaj K, Tornatore C, Sweetser MT, Yang M, Sheikh SI (2012). Placebo-controlled phase 3 study of oral BG-12 for relapsing multiple sclerosis. N Engl J Med.

[8] Kappos L, Radue EW, O'Connor P, Polman C, Hohlfeld R, Calabresi P, Selmaj K, Agoropoulou C, Leyk M, Zhang-Auberson L (2010). A placebo-controlled trial of oral fingolimod in relapsing multiple sclerosis. N Engl J Med.

[9] Kappos L, Wiendl H, Selmaj K, Arnold DL, Havrdova E, Boyko A, Kaufman M, Rose J, Greenberg S, Sweetser M (2015). Daclizumab HYP versus interferon Beta-1a in relapsing multiple sclerosis. N Engl J Med.

[10] O'Connor P, Wolinsky JS, Confavreux C, Comi G, Kappos L, Olsson TP, Benzerdjeb H, Truffinet P, Wang L, Miller A (2011). Randomized trial of oral teriflunomide for relapsing multiple sclerosis. N Engl J Med.

[11] Polman CH, O'Connor PW, Havrdova E, Hutchinson M, Kappos L, Miller DH, Phillips JT, Lublin FD, Giovannoni G, Wajgt A (2006). A randomized, placebo-controlled trial of natalizumab for relapsing multiple sclerosis. N Engl J Med.

[12] Polman CH, Reingold SC, Edan G, Filippi M, Hartung HP, Kappos L, Lublin FD, Metz LM, McFarland HF, O'Connor PW (2005). Diagnostic criteria for multiple sclerosis: 2005 revisions to the “McDonald criteria”. Ann Neurol.

[13] Balak DM, Hengstman GJ, Cakmak A, Thio HB (2012). Cutaneous adverse events associated with disease-modifying treatment in multiple sclerosis: a systematic review. Mult Scler.

[14] Costello K, Kennedy P, Scanzillo J (2008). Recognizing nonadherence in patients with multiple sclerosis and maintaining treatment adherence in the long term. Medscape J Med.

[15] Ziemssen T, Bajenaru OA, Carra A, de Klippel N, de Sa JC, Edland A, Frederiksen JL, Heinzlef O, Karageorgiou KE, Lander Delgado RH (2014). A 2-year observational study of patients with relapsing-remitting multiple sclerosis converting to glatiramer acetate from other disease-modifying therapies: the COPTIMIZE trial. J Neurol.

[16] Metz LM, Patten SB, Archibald CJ, Bakker JI, Harris CJ, Patry DG, Bell RB, Yeung M, Murphy WF, Stoian CA (2004). The effect of immunomodulatory treatment on multiple sclerosis fatigue. J Neurol Neurosurg Psychiatry.

[17] Coyle PK (2016). Management of women with multiple sclerosis through pregnancy and after childbirth. Ther Adv Neurol Disord.

[18] Buron MD, Chalmer TA, Sellebjerg F, Frederiksen J, Gora MK, Illes Z, Kant M, Mezei Z, Petersen T, Rasmussen PV (2019). Comparative effectiveness of teriflunomide and dimethyl fumarate: a nationwide cohort study. Neurology.

[19] Ziemssen T, Calabrese P, Penner IK, Apfel R (2016). QualiCOP: real-world effectiveness, tolerability, and quality of life in patients with relapsing-remitting multiple sclerosis treated with glatiramer acetate, treatment-naive patients, and previously treated patients. J Neurol.

[20] Prosperini L, Gianni C, Leonardi L, De Giglio L, Borriello G, Galgani S, Pozzilli C, Gasperini C (2012). Escalation to natalizumab or switching among immunomodulators in relapsing multiple sclerosis. Mult Scler.

[21] Gajofatto A, Benedetti MD (2015). Treatment strategies for multiple sclerosis: when to start, when to change, when to stop?. World J Clin Cases.

[22] Comi G, De Stefano N, Freedman MS, Barkhof F, Polman CH, Uitdehaag BM, Casset-Semanaz F, Hennessy B, Moraga MS, Rocak S (2012). Comparison of two dosing frequencies of subcutaneous interferon beta-1a in patients with a first clinical demyelinating event suggestive of multiple sclerosis (REFLEX): a phase 3 randomised controlled trial. Lancet Neurol.

[23] Jacobs LD, Beck RW, Simon JH, Kinkel RP, Brownscheidle CM, Murray TJ, Simonian NA, Slasor PJ, Sandrock AW (2000). Intramuscular interferon beta-1a therapy initiated during a first demyelinating event in multiple sclerosis. CHAMPS Study Group. N Engl J Med.

[24] Kappos L, Freedman MS, Polman CH, Edan G, Hartung HP, Miller DH, Montalban X, Barkhof F, Radu EW, Bauer L (2007). Effect of early versus delayed interferon beta-1b treatment on disability after a first clinical event suggestive of multiple sclerosis: a 3-year follow-up analysis of the BENEFIT study. Lancet.

[25] Herbstritt S, Langer-Gould A, Rockhoff M, Haghikia A, Queisser-Wahrendorf A, Gold R, Hellwig K (2016). Glatiramer acetate during early pregnancy: a prospective cohort study. Mult Scler.

