# Supplementary material for: Use of glatiramer acetate between 2010–2015: effectiveness, safety and reasons to start GA as first or second line treatment in Swiss multiple sclerosis patients
Source: BMC Neurol. 2019 Jul 12;19:159. doi: 10.1186/s12883-019-1383-6 (PMC6626416; doi:10.1186/s12883-019-1383-6)
Supplement: Supplementary file 1 — Comparison between the baseline characteristics of patients monitored (V1 V2) and those who had no data on V2 (noV1 V2). (DOCX 15 kb) [file 12883_2019_1383_MOESM1_ESM.docx]

**Additional file 1:** Comparison between the baseline characteristics of patients monitored (V1V2) and those who had no data on V2 (noV1V2)

| **Variables** | | | **V1V2 (n=194)** | **noV1V2 (n=32)** | ***p* (χ^2^/MW)** |
| --- | --- | --- | --- | --- | --- |
| Age [years (IQR)] | | | 38.0 (29.0-46.0) | 36.5 (27.7-46.2) | 0.591 |
| Sex | | F [n (%)] | 139 (71.6) | 21 (65.6) | 0.278 |
|  | | M [n (%)] | 38 (19.6) | 10 (31.3) |  |
|  | | NA [n (%)] | 17 (8.8) | 1 (3.1) |  |
| Disease course | | CIS [n (%)] | 36 (18.6) | 5 (15.6) | 0.879 |
|  | | RRMS [n (%)] | 158 (81.4) | 27 (84.4) |  |
| Age at onset [years (IQR)] | | | 31.0 (25.0-40.0) | 33.0 (24.2-43.0) | 0.657 |
| Disease duration [years (IQR)] | | | 3.0 (0.0-9.0) | 2.0 (0.0-5.7) | 0.492 |
| Treatment naïve | No, n (%) | | 64 (33.0) | 7 (21.9) | 0.294 |
|  | Yes, n (%) | | 130 (67.0) | 25 (78.1) |  |
| ARR previous 24 months [n (IQR)] | | | 0.5 (0.5-1.0) | 0.5 (0.5-1.0) | 0.268 |
| EDSS at V1 [score (IQR)] | | | 2.0 (1.5-3.0) | 2.0 (1.5-3.0) | 0.751 |

ARR annualized relapse rate; CIS Clinically isolated syndromes; EDSS Expanded disability status scale; IQR inter quartile range; RRMS relapsing remitting multiple sclerosis
