# Supplementary material for: Use of glatiramer acetate between 2010–2015: effectiveness, safety and reasons to start GA as first or second line treatment in Swiss multiple sclerosis patients
Source: BMC Neurol. 2019 Jul 12;19:159. doi: 10.1186/s12883-019-1383-6 (PMC6626416; doi:10.1186/s12883-019-1383-6)
Supplement: Supplementary file 2 — Adverse events reported during study period. (DOCX 14 kb) [file 12883_2019_1383_MOESM2_ESM.docx]

**Additional file 2:** Adverse events reported during study period

| **Patients with adverse events** | **N**  **(n=26)** |
| --- | --- |
| Overall adverse event episodes | 28 |
| Injection site reactions | 7 |
| Relapses | 4 |
| Post injection systemic reaction | 2 |
| Pregnancy | 8 |
| EDSS worsening | 1 |
| Psoriasis | 1 |
| Hypotension | 1 |
| Palpitations | 1 |
| Allergic reaction | 1 |
| Lymphadenopathy | 1 |
| Increased liver enzymes | 1 |
| Muscle cramps | 1 |
