# Supplementary figures and images for: Use of glatiramer acetate between 2010–2015: effectiveness, safety and reasons to start GA as first or second line treatment in Swiss multiple sclerosis patients
Source: BMC Neurol. 2019 Jul 12;19:159. doi: 10.1186/s12883-019-1383-6 (PMC6626416; doi:10.1186/s12883-019-1383-6)

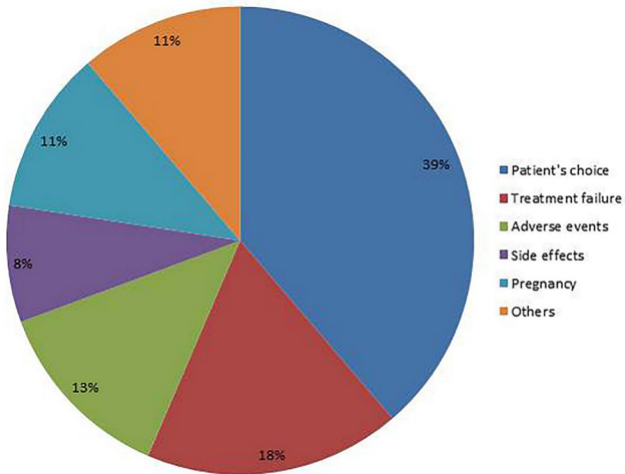

Supplement: Supplementary file 3 — Reasons for discontinuing GA treatment before V2 (N = 62). (PDF 572 kb) [file 12883_2019_1383_MOESM3_ESM.pdf]
